# A digital and nurse-led support intervention, first year after prostate cancer treatment: a single-arm feasibility study in a Swedish primary care setting

**DOI:** 10.1186/s12875-024-02669-x

**Published:** 2024-12-03

**Authors:** Nazmije Kelmendi, Ann Langius-Eklöf, Marina Taloyan, Kay Sundberg, Åsa Craftman, Marie Nilsson

**Affiliations:** 1 Department of Neurobiology, Care Sciences and Society, Division of Nursing, Karolinska Institutet, Alfred Nobels Allé 23, Stockholm, 141 52 Sweden; 2 Academic Primary Health Care Center, Region Stockholm, Stockholm, 113 65 Sweden; 3 Department of Neurobiology, Care Sciences and Society, Division of Family Medicine and Primary Care, Karolinska Institutet, Alfred Nobels Allé 23, Stockholm, SE-141 52 Sweden

**Keywords:** Prostate cancer, Primary care, Feasibility, Intervention, Nurse, Self-management, Follow-up, Support, Digital health, eHealth

## Abstract

**Background:**

The prevalence of patients with prostate cancer is increasing, and the first year after treatment is a vulnerable period for patients as they experience symptoms and unmet needs. Although electronic patient-reported outcomes that focus on patient self-management have demonstrated benefits, evidence is sparse regarding patients with prostate cancer in primary care settings and the role of nurses as a supportive resource. The significant role of primary care in future cancer care is on the agenda. This study aims to test the feasibility of a complex intervention that includes electronic patient-reported outcomes and self-management advice in an app, combined with nurse-led support, in primary care settings during the first year after curative intended prostate cancer treatment.

**Methods:**

The intervention lasted four weeks and was a single-arm study. Feasibility was assessed by examining the recruitment process, retention rate, adherence to the reporting of symptoms in the app, and acceptability of the intervention. Data was collected through (1) logged data from the app that included patient-reported outcomes and self-management advice, (2) field notes by the nurse, and (3) semi-structured interviews with patients. Descriptive statistics were applied to logged data. The interviews and the field notes were analysed using qualitative content analysis.

**Results:**

The recruitment rate was 55%, yielding 11 patients with high retention as all completed the intervention. Adherence to reporting was 100%, and all functions in the app were used. Individual variation in how patients used the app was found, which was attributed to patients’ current needs. In total, 36 health dialogues with the nurse (virtual, face-to-face, telephone) were performed; all first dialogues lasted longer, while follow-ups were shorter. Patients described that the health dialogues covered relevant subjects and that the combination of using the app and health dialogues was tailored and provided supplementary support. No adverse events occurred; however, a few technical difficulties interfered with the intervention, and the patients gave valuable suggestions for improvement. Furthermore, patients suggested that the intervention should start immediately after treatment.

**Conclusion:**

As the patients adhered to and accepted the intervention, it was considered feasible. Findings suggest intervention should start directly after treatment ends.

## Background

Prostate cancer (PC) is the second most common cancer in men worldwide with a rising prevalence [1]. After ending treatment, patients may experience various psychological and physical symptoms, such as urinary, bowel and sexual dysfunctions, decreased quality of life [2], anxiety and depression, and fear of cancer recurrence [3]. Furthermore, patients report unmet needs, such as a lack of information and support from healthcare [4]. The first year after treatment during follow-up is a vulnerable period, where patients testify to being left to rehabilitate themselves without sufficient support from healthcare while still suffering from physical and psychological side effects [5, 6].

Due to the expected increase in the number of cancer survivors, it is suggested that existing pathways of follow-up care after cancer treatment need to be redesigned so the healthcare system has the resources to provide sufficient support to patients [7–9]. Different types of models of care for cancer survivors led by primary-care providers, oncology nurses, or those with shared responsibility between oncology care and primary care setting, have shown to be at least as effective as specialist-led care [8, 10, 11]. The significant role of primary care in future cancer care is on the agenda for many countries and there is a growing recognition that primary care can take a more active role [8, 9, 12–14].

In the Swedish context, policy documents highlight the role of primary care in facilitating coherent care chains which cover health promotion, investigation, diagnosis, follow-up and rehabilitation [15]. Furthermore, the Swedish government has proposed a significant shift in the overall healthcare organisation, with a focus on person-centred and integrated care in primary care [16]. At the time of this study, the follow-up in the first year after curative treatment for prostate cancer primarily occurs within the specialist care setting. The follow-ups consist of regular prostate-specific antigen (PSA) measurements, an appointment two to three months after PC treatment ends, and beyond that, patients have access to a contact nurse when needed [17]. Patients with cancer in Sweden tend not to consult primary care for assistance with their treatment-related side effects [18].

Follow-ups after cancer treatment are suggested to include electronic patient-reported outcomes (ePROs) to identify patients’ needs and improve communication between different stakeholders [19, 20]. Studies have shown feasibility and acceptability of apps that collect ePRO during treatment for prostate cancer [21, 22]. An app that offered self-management support to patients after radical prostatectomy did not give evidence of fewer side effects or enhanced self-care [23]. The combination of ePRO and self-management advice in apps is increasing and showing promising results among patients with cancer [24]. The importance of integrating ePRO and self-management support with professional monitoring is emphasised [19, 25]. Beneficial interventions for self-management need to be multidimensional and incorporate education as well as psychosocial support [26]. A previous study showed the feasibility of such intervention to survivors of prostate cancer approximately two years after treatment in a primary care context, but to increase beneficial outcomes it was suggested that it be implemented earlier [27]. However, we have not found any studies using ePROs as a foundation for self-management among patients with prostate cancer in primary care settings.

The predicted rising number of prostate cancer survivors and their different care needs is expected to increase the demands on healthcare providers to provide cost-effective and person-centred supportive care to patients. The first year after curative treatment is a vulnerable period for the patients and, therefore, an important period to prevent complications and recurrence [5, 6]. Primary care may be an important arena both for patients and the healthcare system for supportive interventions for patients with prostate cancer after curative intended treatment. However further research in primary care context is needed.

### Objectives

This study aims to test the feasibility of a complex intervention that includes electronic patient-reported outcomes and self-management advice combined with nurse-led support in primary care settings during the first year after curative intended prostate cancer treatment.

## Method

### Design

This feasibility study is designed as a single-arm study and based on the Medical Research Councils (MRC) complex intervention evaluation framework, phase two (feasibility and piloting) [28]. The study is descriptive and takes both a qualitative and quantitative approach to achieve a comprehensive perspective. We have followed the CONSORT [29] and the TIDieR [30] checklists.

The intervention (described in detail below) is a combination of using an app (Interaktor) and health dialogues with a nurse in primary care.

Feasibility was assessed by evaluating the recruitment process using a database, and retention rate. Adherence, and acceptability to the intervention were assessed through logged data, semi-structured interviews with the patients and field notes from the nurse.

Pre-specified criteria to evaluate the feasibility of the intervention included: (a) identifying and recruiting 10–15 eligible patients and retaining them for four weeks; (b) adherence to the reporting of symptoms in the app at minimum once a week; and (c) relevance and satisfaction with the intervention, which we considered as indicators of acceptability.

### Setting and participants

The study was performed at two primary care centres (PCCs) in the Stockholm Region, Sweden. There was one study-specific nurse (first author) involved at both PCCs. The nurse had nine years of experience working as a registered nurse and an additional five years as a district nurse in primary care. She had knowledge of oncology, and experience working with patients with cancer in the secondary care setting.

#### Recruitment

A purposive strategy was used to identify patients through the Stockholm Region Council’s administrative database on healthcare consumption (VAL) [31] by combining the PCCs’ individual identification codes with the ICD-code (C61.9 Malignant neoplasm of prostate cancer), procedure codes (radiotherapy, surgery), and date of treatment (< 12 months since treatment). Inclusion criteria were patients who had recently or up to 12 months previously ended curative intent treatment for prostate cancer. Exclusion criteria were not Swedish-speaking and/or having cognitive impairment. Recruitment lasted from February 2021 to May 2021. No patients were excluded. Twenty patients met the inclusion criteria and were invited to participate. Envelopes containing study information and pre-addressed reply envelopes were sent to the identified patients. Eleven patients were included and provided consent for participation.

### Intervention

The intervention was developed before the study started based on interviews with patients, healthcare professionals [6] and existing literature. For four weeks, patients utilised an app called Interaktor to report ePROs and receive self-management advice. They were also engaged in health dialogues with the study nurse. The time frame was deemed sufficient for the patients to test the app and to have the opportunity to have at least two health dialogues.

#### The app interaktor

Interaktor is a non-commercial app developed for research purposes only and has previously been evaluated during treatment for pancreatic, breast, and prostate cancer [32–34]. The app is stored on a safe server at the university and the safety protocol Secure Socket Layer was used for user authentication and encrypted data transfer between the device and server. The content of this app, developed for patients with PC after curative treatment, is based on a standardised symptom questionnaire [35], interviews with patients and healthcare professionals, including a review of the literature [6], and national guidelines [17].

Interaktor includes: (1) symptom questions of occurrence, frequency and distress and a function with the opportunity to write free-text comments; (2) access to information about self-management strategies; (3) graphs for the patients to view their reporting history; (4) an alert system that notifies a nurse of severe symptoms; (5) a reminder function to report; and (6) a web interface for nurses. Interaktor has two interfaces: one for patients and one for health providers.

Patients could download the app on Android or Apple devices or access it from a web address in a browser. This version, developed for patients with PC after curative treatment, includes:

A) Sixteen questions about urine dysfunctions (leakage, difficulties urinating, blood in urine), bowel dysfunctions (constipation, loose stool/diarrhoea, flatulence, blood in stool), worry/anxiety, sadness/depression, sleep, pain, fatigue (including decreased strength), loss of appetite, hot flushes (including sweating), swelling/lymphoedema, and sexual health. All questions, except sexual health, were mandatory and were answered with yes or no. If the patient answered yes, they received two follow-up questions: how often and how distressing, with four grading scale options for respective questions (*not often/sometimes/fairly often/very often*, and *not distressing/a little distressing/fairly distressing/very distressing).*
B) The self-management function contained general information about side effects (what has caused them and a description of how the symptom is experienced), advice about self-management strategies, advice of what symptoms or concerns related to the reported side-effect should promote contact with a healthcare professional, and further information via external linking to reliable documents, webpages, or videos (there were 66 external links). In addition, there was information about lifestyle habits (smoking, alcohol, nutrition, physical activity) and general information about prostate cancer and treatment. After a report is performed, a referral notification is generated for patients to access self-management advice related to symptoms answered with a yes. The patients also had access to self-management advice at any time.
C) Graphs were available as feedback for the patients to view their reporting history.
D) Alerts were generated only for; pain, blood in stool, blood in urine, and loss of appetite when patients answered that the symptom/s occurred *very often* and were *very distressing*. Alerts generated a notification for the patients with the following written information:” *You will be contacted within 24 h. NOTE*: *This only applies on weekdays from 8 am to 3 pm when your nurse is on duty. If you experience acute symptoms*, *seek emergency care”.*
E) The app had a reminder function to make a new report eight days after the last report.

The web interface for the nurse included information about the patient’s name and PCC. The nurse could monitor and sign the incoming patient reports in real-time and view graphs of reported history and available alerts. If an alert was generated, the nurse received a notification on her phone or computer to contact the patient and plan additional actions.

The patients were instructed to report their symptoms preferably twice a week and at least once a week, and to read the self-management advice in the app.

#### The health dialogues

The health dialogues were tailored to patients’ needs based on the patient-reported assessments of symptoms and concerns. The health dialogues were also based on the nurse’s suggestions, and motivational interviewing (MI); a person-centred conversation style intended to promote behavioural change regarding various problems [36].

Patients could choose between telephone, virtual, or physical appointments. The study nurse booked an initial health dialogue after receiving signed consent, provided access to the app and written and verbal instructions for downloading it, monitored the incoming reports during office hours, and booked a telephone follow-up two weeks after the first health dialogue. Except for these instructions, there was no pre-determined length or number of health dialogues, as the intervention was designed to be tailored to patients’ individual needs.

### Data collection

#### Logged data from the app

Logged data included the total number of reports, reported symptoms, free-text comments, viewed self-management advice, viewed external links to webpages and general information, viewed graphs, and alerts. The logged data were extracted at Karolinska Institutet (KI) as an encrypted Excel file from the database hosted on the secure KI server, and the data were available at individual and group levels.

#### Field notes from the nurse

The nurse wrote field notes after each contact with the patients (*n* = 36) in a document pre-designed for the study. It included the following headlines: technical issues, type of meeting (telephone, virtual, physical), workload (time consumption per appointment), and a free text option for other thoughts and concerns during the intervention.

#### Semi-structured interviews with patients

The last author performed semi-structured telephone interviews between March and July 2021, after the patients had completed the four-week intervention. The interviews lasted, on average, 29 min (range: 19–55 min). Ten of 11 interviews were recorded. One patient did not want to be recorded, so the last author took notes during the interview. The interviews followed a semi-structured interview guide developed for the study regarding patients’ experiences of the intervention (Table 1). The interview guide had follow-up questions specifically regarding the content of the app and the health dialogues. All interviews were transcribed verbatim by the first author.

**Table 1:** Interview guide with patients

Questions	Follow-up questions
How did you experience using the app and its different features?	What did you think about…? Reporting/answering questions? Self-management advice? Graphs/statistics? How did the technical elements work (downloading, login, instructions)?
What was it like to have health dialogues with a nurse regarding your prostate cancer?	How did you feel about the advice? How did you use the advice? Anything missing? Anything you wished had gone differently?
How did you experience using the app and having health dialogues with a nurse?	
Is there anything else you want to add that we have not discussed?	

### Data analysis

#### Logged data from the app

The extracted logged data from Interaktor were analysed using descriptive statistics in Microsoft^®^ Excel^®^ for Microsoft 365 MSO (Version 2407 Build 16.0.17830.20166). Adherence was assessed by counting the frequency of patient reports according to the pre-determined instructions, at least once a week.

#### Field notes from the nurse

The number and type of appointments (physical, telephone, virtual) and information about the time consumption were analysed using descriptive statistics. The nurse’s free-text notes were analysed by the last author using manifest content analysis, without creating categories or themes, and thereafter discussed and refined with all authors.

#### Semi-structured interviews with patients

The first and the last authors analysed the transcribed interviews using inductive qualitative content analysis [37]. This method was chosen to draw valid conclusions about a manifest message by systematic identification. First, the authors read the interviews individually to comprehensively understand the whole. Thereafter relevant text was extracted, using an open coding process. Identified codes were transferred onto a coding sheet and organised into subcategories based on similarities and differences. The subcategories were revised and grouped into broader generic categories. Finally, all authors discussed refinement and categorising.

## Results

### Recruitment and participant

Eleven patients agreed to participate (recruitment rate 55%). The 11 included patients were aged 57 to 75 (mean 66, median 68) and further descriptive data are provided in Table 2. All included patients completed the four-week intervention, meaning no dropouts occurred.

**Table 2:** Sociodemographic, clinical characteristics, and treatment of patients (*n* = 11)

	Patients (*n* = 11)
**Age** (range 57 to 75 years, mean 66, median 68)	
≤ 60	1
≤ 65	3
≤ 70	5
≤ 75	2
**Partnership**	
Single	2
Married or having a partner	9
**Education level**	
College (secondary school)	7
University	4
**Employment**	
Partially retired/employed	1
Retired	6
Employed	4
**Treatment**	
Radical prostatectomy	7
Radiotherapy/Brachytherapy	3
Radiotherapy combined with hormonal treatment	1
**Time since the end of treatment** (months)	
≤ 3	5
≤ 6	2
≤ 9	1
≤ 12	3
**Comorbidities**	
Yes	10
No	1

### Logged data

**Fig. 1:**
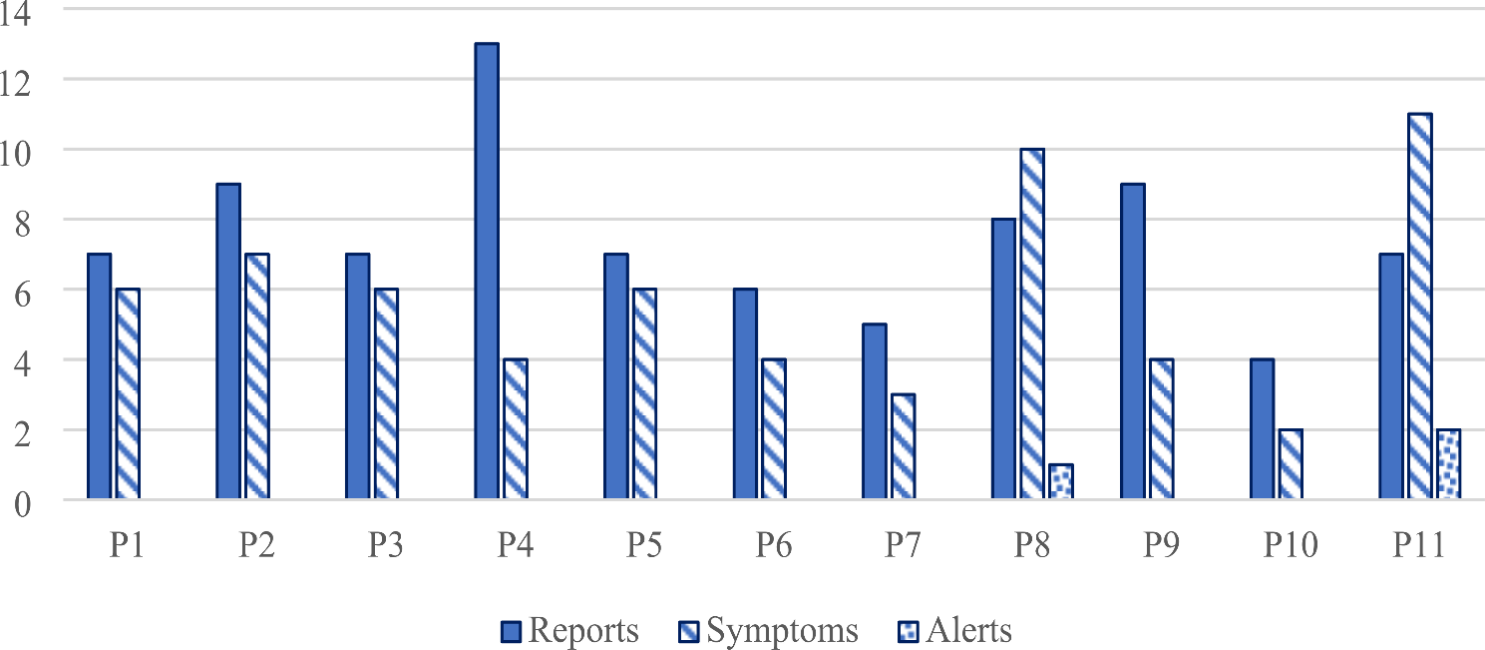
The total number of reports, symptoms, and alerts per patient (P1-P11)

The logged data show that all features of the app were utilised. On a group level, a total of 82 reports were performed (median 7, range 4–13 reports/participant). Thus, all patients reported at least once a week during the four-week intervention. In total 244 symptoms were reported (median 6, range 2–11 symptoms/participant) (Fig. 1).

Three alerts were generated, but all three were false due to a misconfiguration in the app. Fourteen of the 16 (87.5%) questions regarding symptoms were reported; no one reported the symptoms *blood in urine* or *blood in stool.* Decreased sexual health was the most reported symptom and the only symptom reported by all patients, whereas loss of appetite was the least reported symptom (Table 3).

**Table 3:** The total number of reported symptoms and viewed self-management advice per symptom at the group level

Type of symptom (in descending order)	Reported symptoms		Viewed self-management advice	
	Number of total reported symptoms (*n* = 244)	Number of patients reporting the symptom	**Number of viewed self-management advice** (including external links) (*n* = 77)	Number of patients viewing the self-management advice
Sexual health	59	11	21	8
Urine dysfunction − leakage	28	6	8^a^	4^a^
Fatigue/Decreased strength	24	6	1	1
Sadness/Depression	20	4	4	4
Flatulence	18	6	9	5
Concerns/Anxiety	15	2	0	0
Pain	13	5	2	2
Sleep disturbance	13	4	1	1
Hot flushes/Sweating	13	3	5	2
Constipation	11	3	3	1
Swelling/Lymphedema	10	3	9	2
Urine dysfunction − difficulties urinating	9	3	8^a^	4^a^
Loose stool/diarrhoea	8	4	5	3
Loss of appetite	3	2	0^b^	0^b^
Blood in stool	0	0	1	1
Blood in urine	0	0	0	0

a: One document for all types of incontinence

b: No advice concerning this symptom

Four patients (36%) used the free-text component on five occasions (one used it twice). The comments gave an additional explanation regarding resolved constipation, sleep disturbance due to increased nightly urinating, pain from surgery scar, flatulence, and impotence. Three (27%) patients used the free-text option regarding the sexual health question 10 times in total concerning non-effective medications for impotence.

**Fig. 2:**
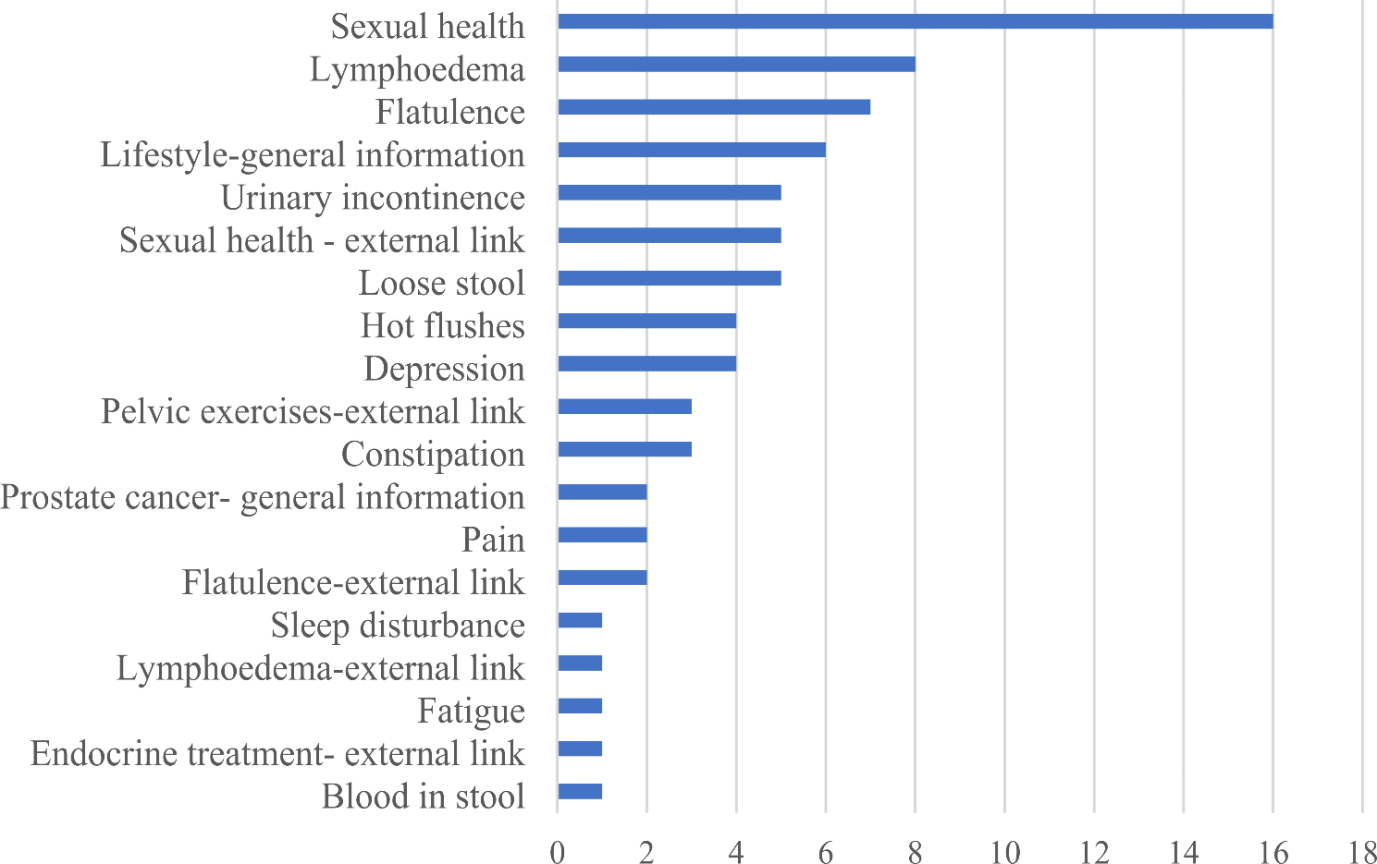
The total number of views at group level regarding self-management advice and their external links, information about *Lifestyle in general*, and *Prostate cancer in general*

All self-management advice was viewed by the patients (median 5, range 1–10 per patient) except for blood in urine and anxiety; the most viewed self-management advice concerned sexual health, followed by lymphoedema (Fig. 2). Five of 66 external links were viewed. The graphs of reported history were viewed by nine (81.8%) of the 11 patients a total of 31 times (median 2, range 1–12 views per patient). The three false alerts and 10 free-text comments regarding decreased sexual health were notified to the patients but not in the nurse version.

### Field notes

**Fig. 3:**
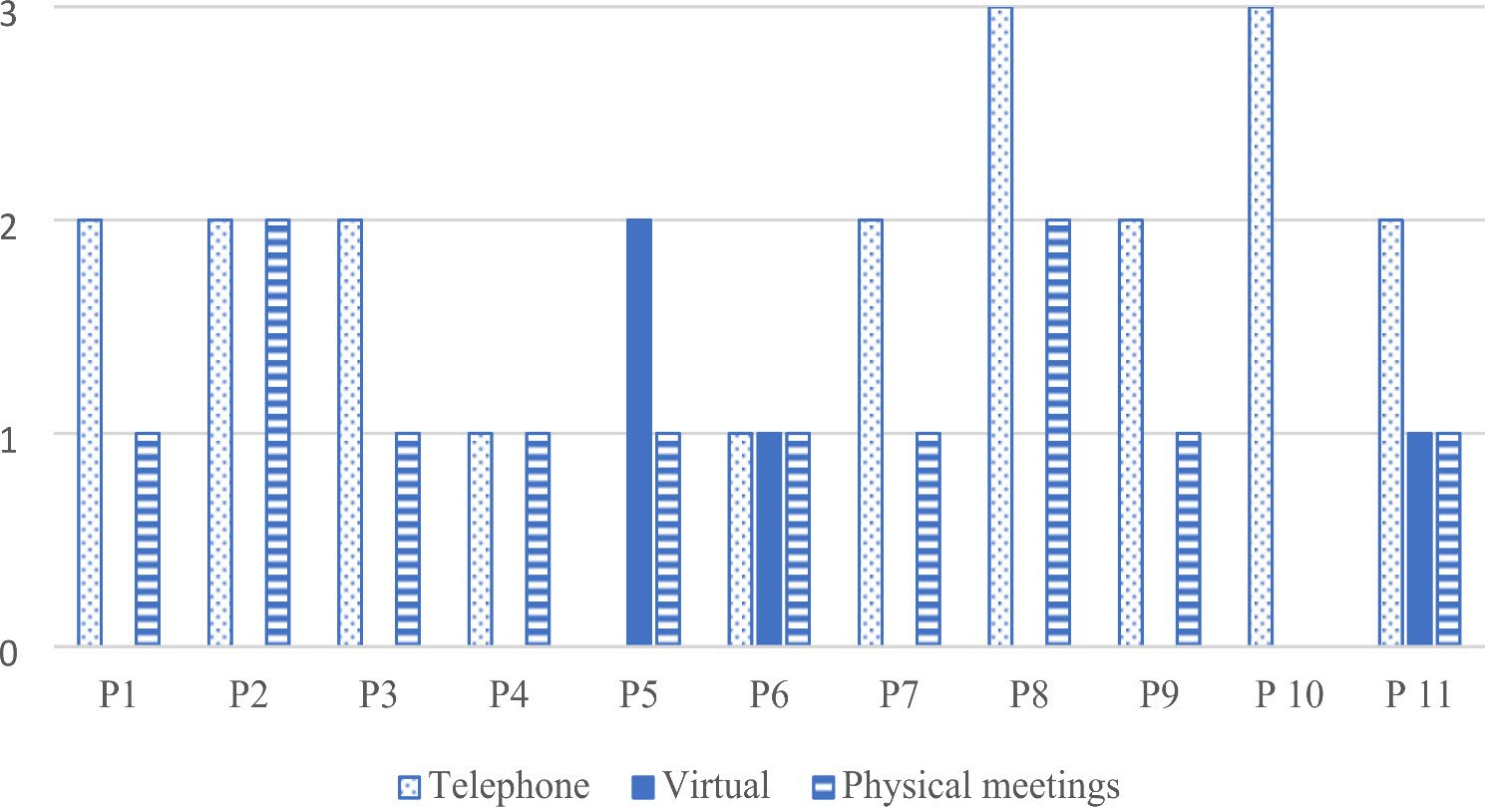
Number of contacts with the nurse during the four-week intervention per patient (P1-P11)

There were, in total, 36 health dialogues with all the patients (Fig. 3). All patients, except one, had at least one physical appointment during the study. This patient avoided going out in public due to the ongoing pandemic. At the first health dialogue with the nurse, nine patients chose a physical appointment, one a telephone, and one a virtual appointment. All first appointments with the patients, irrespective of form, lasted on average 60 min (range 55–90). The nurse noted some technical problems; most were concerned with downloading the app, which interfered with the first health dialogues by shifting focus to technical issues and making those encounters last longer. Three patients choose to access the app through the web address. The app worked as intended once any technical issues were resolved with a technician. The follow-up appointments were, in general, shorter than the first. The physical follow-up meetings lasted 48 min on average (range 35–60), the telephone follow-up calls, 16 min (range 5–60), and the virtual appointments, 35 min (range 30–45).

Several topics were raised during the health dialogues; these were side effects after cancer treatment, urine incontinence, constipation, pain, lymphoedema, sadness/depression, and decreased sexual health. Additionally, nutrition and physical activity were discussed. Common actions for the nurse included prescribing medication for constipation and different aids for incontinence protection, providing patients with information and making referrals to other healthcare professionals when necessary.

The nurse suggested refinements to the app, regarding, for example, the log-in procedure and downloading, including a notification for the patient when the nurse had viewed and signed a report, and more options for the nurse version when signing the reports. Further suggestions were more free-text options for some symptoms, e.g., pain, so that the patients could clarify their symptoms. The study was performed during the Covid-19 pandemic, which rendered new digital ways to communicate with patients. The nurse noted the benefit of the possibility to adjust communication methods, such as utilising telephone or virtual appointments for health dialogues.

### Interviews with patients

The analysis of the interviews rendered 3 generic categories: *Flexible and tailored support*, *Individual usage of the intervention* and *Suggestions for improvement*, with eight, five and four sub-categories, respectively (Table 4).

**Table 4:** Generic categories and sub-categories

Generic category	Sub-category
**Flexible and tailored support**	Relevant app questions The app’s features provide an overview Quality-assured and updated information in the app Simple reporting Flexible meetings The combination adds value Expanded knowledge and practical help Helpful and supportive nurse
**Individual usage of the intervention**	Patient’s role in the health dialogues Reading advice and following links Positive about free text/self-expression Individualised planning and follow-up App usage guided by needs
**Suggestions for improvement**	Desire to clarify answers with comments Request for expanded reporting Desire for more information Request for simpler download and login

#### Flexible and tailored support

Patients described the intervention as valuable in various ways. They expressed that this type of intervention should start immediately after treatment, and those patients who gained access to it directly after treatment stated that they were satisfied with the current time frame.

Most patients described the app and the health dialogues as tailored to their current needs since the combination of those two provided supplementary support. Repeated support and information, and from several sources, gave the patients explanations, increased knowledge, and awareness of their occurring symptoms. Some patients expressed that it was easy to forget things they wanted to discuss in the health dialogues, therefore, it was appreciated that the nurse could follow their reported symptoms in the app and use them as a basis for the health dialogues.

*“It’s a good combination because I can report when it suits me in my own time and think over what problems I’ve had. Then*, *the report becomes a basis for discussion and feedback for the occasions when you meet. So*, *I think it’s an ideal combination. Otherwise*, *it is easy to forget things in a meeting.” (P5)*.

The information in the app was generally perceived as relevant, easy to access, quality-assured, and up to date. However, the patients who did not have Swedish as their native language found some words challenging. Patients appreciated the opportunity to receive information and advice about their reported symptoms, which was also described as useful and safe, instead of searching for information elsewhere. The regular reporting was voiced as allowing time for reflection and increased awareness of symptoms such as lymphoedema, and the graphs clarified the symptom progress over time. The health dialogues were experienced as informative, covering relevant subjects, and providing additional support. This was exemplified by information regarding the physical recovery process, symptoms, practical support regarding incontinence protection and pelvic exercises, medication prescriptions, and follow-ups. Patients stated that the obtained information and self-management advice from both the app and the nurse were, in general, new, but that it also reminded them of previously received information. Receiving information repeatedly was described as meaningful, which could be helpful in the future.

*“To read the advice on what to do*, *I thought that was superb. To be able to get it delivered*, *otherwise I wouldn’t have read it. You can search for information on the internet*, *1177* (national Swedish service that provides healthcare information and advice) *or something like that*, *but if I didn’t open the computer and search for it myself*, *then you leave it. If I get a notification*, *I get the advice right away. I think that is superb.” (P7)*.

Regardless of the appointment type, patients expressed that the app and the health dialogues were proactive, interactive, and efficient ways of receiving care, which they thought could also lessen the burden on healthcare. An initial physical meeting was preferred as it developed an alliance between the nurse and the patient. Additionally, patients appreciated the flexibility of the health dialogues in terms of length and form, including physical meetings, telephone calls, and virtual meetings. The virtual meetings made it easier to contact the nurse and plan appointments. The patients perceived the health dialogues as motivating and caring. Furthermore, they expressed a feeling of being safe; this was exemplified by patients being able to talk about sensitive subjects such as sexual health, that the nurse motivated them to act in situations of symptom distress, knowing that the nurse would call if there were an alert and that the nurse was available at their PCC in case of any complication.

Patients described the app as mostly relevant regarding the time interval for symptom reporting frequency, symptom questions, self-management advice, information, and the opportunity to formulate and express other potential needs and questions in the free-text comments. The reminder function (if the patient had not made a report) and referral function (to read self-management advice related to the patient’s reported symptoms) were also appreciated.

#### Individual usage of the intervention

Patients with low symptom burden felt less need to use the app. Some patients did not feel motivated to use the graphs, although they acknowledged that the history of symptoms visualised in the graphs could be helpful in the longer term. Even though some questions were deemed irrelevant on some reporting occasions, the patients expressed that these questions should be retained since symptoms may change over time and become relevant. Patients voiced how the use of self-management advice varied; some read only the parts they found relevant, others read all the advice, and some applied the advice they received. Other patients said they had read the advice but did not follow it despite having symptom distress, and some described not reading the advice because they did not experience symptom distress or found themself already well-informed. Furthermore, some patients described how they had not followed the self-management advice they had received from the nurse. Patients pointed out that they are also responsible for following self-management advice, and regarding what to bring up in the health dialogues. One patient described wanting more appointments with the nurse due to concerns but never raised this question during the health dialogues.

*“It was good advice; we talked about sexuality and things like that. Then she advised me to reach out to my contact nurse regarding that. I have not done that…I have to pull myself together and talk to the cancer nurse.” (P1)*.

#### Suggestions for improvement

A few patients experienced the app, and technology in general, as complex, specifically regarding downloading the app. Patients expressed that the log-in procedure was complicated as they had to insert their email and password each time.

Some patients suggested expanding the information and opportunities for communication and reporting. The suggestions were to include a two-way chat function, the ability to clarify answers with free text, and other reminder notifications, for example, to do their pelvic exercises. Other requests were information about other urine-related symptoms, surgical scars, catheter, and incontinence pad pictures in the app. One patient wanted access to blood tests results in the app.

## Discussion

This study indicates that a primary care-based intervention, combining nurse-led supportive care with an app for symptom reporting and self-management advice, to be feasible in a group of patients with prostate cancer in their first year after treatment. The present intervention was built on suggestions from other studies showing that effective interventions should combine education with psychosocial support [26]. Unlike most studies that are based in the secondary care context, this intervention was tested within a primary care context with patient entry during the first year after treatment ends. It was suggested by some patients that the intervention should be initiated immediately after treatment.

The recruitment rate for our study (55%) was higher than reported in a web-based feasibility study (13–22%) focusing on holistic needs [38] and slightly lower than reported in a pilot study (61%) [27]. A systematic review of online supportive care interventions for men with prostate cancer described an average recruitment rate of 54% in 15 studies (ranging from 5 to 95%) and an average retention rate of 78% [39]. Our findings showed a higher retention rate for the intervention, as no patients dropped out during the study. Patient retention is crucial for research studies, as high dropout rates affect the study’s validity and generalizability.

The combination of reporting symptoms in the app and discussing them in the health dialogues with the nurse was appreciated by patients and described as receiving tailored and supplementary support and information. The relevance of app Interaktor was shown by being frequently used i.e. high adherence according to given instructions. All patients reported at least once a week some even more than instructed. Furthermore, the flexibility of the app was reflected by the patients’ own choice to take an active role in how to use the different components in the app, indicating that the app facilitates individualised care. Using the app Interaktor during radiotherapy for prostate cancer has earlier shown high adherence and acceptability [33, 40], and rendered less symptom burden at the end of treatment compared to a control group [40]. Other studies using electronic systems for collecting patient-reported outcomes have shown usage adherence with a variation between 45 and 92% [41]. Interactivity and individualisation were factors that facilitated patient engagement. In a recent study patients with prostate cancer received digital rehabilitation support without physical contact with health care and after one year the usage had decreased markedly to 11% [23]. In a four-arm RCT study, patients who received additional coaching in the form of human interaction were generally more satisfied and more successful in making improved lifestyle changes than the other groups [42]. The patients in our study emphasised the importance of the initial physical meeting (health dialogue) with the nurse as it played a valuable role in establishing and developing an alliance. One example is that the intervention seemed to provide an opportunity for patients to raise issues concerning sexual health, which usually is described as a sensitive topic to talk about [6].

The patients, in this study, gave the impression of being motivated to take an active role in managing their health through encounters in the health dialogues and using the app. In line with other studies [40, 43, 44] the patient’s own needs and preferences determined their overall app usage. Very few external links were used, which may reflect a sufficient content of the self-management advice within the app. The most viewed self-management advice was concerning sexual health and lymphoedema, also common topics in the health dialogues. Lymphoedema has been noticed as an underestimated condition in this group of patients [45] and not included in questionnaires evaluating side effects one year after treatment [2]. Interestingly, fatigue was commonly reported in the app, but the self-management advice was not read to a great extent in the app or noted in the field notes as a topic brought up in the health dialogues.

Some patients voiced how they, despite receiving advice from the app and the nurse, did not follow the self-management recommendations. The challenge to motivate patients with prostate cancer to act on recommendations has been described earlier [27]. It is important that healthcare professionals ascertain that patients have understood the information they have been given and validate patients’ concerns and different needs for support. Health behaviour changes and symptom improvements take time [46], and motivating patients is principal in facilitating these changes [47]. When working on changes in health behaviour, it’s essential to address them with combinations of different approaches, including team collaboration, scheduling follow-up visits, and assessing progression in self-management [46].

The balance between standardised and tailored support approaches is essential to ensure so that patients receive the best possible care. Several patients described the advantages of standardised approaches and wanted more reminders in the app to report symptoms or perform pelvic exercises. In contrast, some patients expressed a desire to have more opportunities to elaborate their symptoms in free text and to chat with healthcare providers, which enables the identification of needs and symptoms other than those in the present app. For example, all symptoms in the app were reported at some time during the study except for blood in urine and blood in stool. These symptoms are less common side effects following curative treatment, but they could also serve as potential indicators of signs of cancer recurrence [48], and are therefore important questions to keep in the app. The patients described that they understood that several features in the app were needed even though they were not relevant to them at that moment but could be in the future.

Online supportive interventions for patients with prostate cancer show promising outcomes [39, 49, 50]. However, these studies are associated with limitations related to, for example, adherence, and heterogeneity in sampling and/or resources. We have only found one study showing feasibility of an intervention for patients with prostate cancer within a primary care setting [27], despite the highlighted role of primary care in future cancer care. The level of primary care involvement in cancer care varies across countries [51]. In Sweden, patients rarely contact primary care regarding their treatment-related side effects [18], and patients are unsure of oncology competence in primary care [18, 51]. In the Netherlands and Canada prostate cancer survivors seem to have more contact with their general practitioner during cancer follow-up [52, 53]. In a study in England, the nurses at primary care centres received structured education in prostate cancer care but still expressed uncertainty in their knowledge. They described this as related to the low number of prostate cancer patients in each centre, and long gaps between appointments, which led to problems in keeping updated [27]. The complexity of cancer care needs, e.g. side effects after different treatments, indicates a need for education on evidence-based knowledge in oncology and rehabilitation. The importance of primary care providers to possess cancer competence (cancer survivorship epidemiology, monitoring for cancer recurrences, screening and managing long-term and late effects of cancer, and promoting overall health) has previously been highlighted, especially due to the increasing number of cancer survivors [9]. Such information is already available and accessible in Sweden through web-based clinical practical guidelines [54]. However, available information does not ensure increased knowledge and accurate implementation in practice. It is important that this information is combined with, for example, educational efforts for health care professional in form of pre-recorded seminars and workshops [9].

In the present study, patients expressed satisfaction with both the length and number of appointments. The patients had the option to receive an unlimited number and length of health dialogues which might have been difficult to fulfil in a regular setting. Notably, the duration of visits in our study aligns with a similar study [27]. The time utilisation in primary care is essential [55] as it determines the number of patients health care staff can accommodate during a working day. This study highlights the potential benefit of collecting ePRO in primary care, as healthcare providers can tailor the length and frequency of appointments based on needs, and health resources might be used more effectively. Moreover, the flexibility in appointment scheduling could be particularly beneficial for patients with limited access to healthcare due to rural areas and geographical circumstances [56].

The results provide valuable insights for improvements, such as an easier way to download the application, easier log-in, and a more visible free text option. Some technical errors occurred, such as false alerts; however, patients did not report/describe any adverse health effects during the intervention. Further improvement suggestions are that patients receive feedback when the nurse has read the reports which would provide additional security and interaction. Further research should focus on evaluating the intervention effects on symptom burden and quality of life among patients with prostate cancer and to determine if early detection of symptoms can prevent further illness. The intervention can be performed in the primary care context, although it is important that the nurses have sufficient knowledge. An effectiveness study should be combined with a process evaluation to especially identify potential barriers in primary care settings [57].

### Strengths and limitations

The results should be interpreted with caution due to the short intervention period and the small sample size.

One strength of this study is its use of both qualitative and quantitative approaches, which provides a comprehensive understanding. By combining both approaches, data can be triangulated, which increases reliability and validity due to a more complete and nuanced understanding [58]. A strength of recruiting from a database is the absence of gatekeepers and selection bias, which according to a systematic review was a consistent issue in online supportive interventions for men with PC [39]. The participants varied in age, time after treatment, and type of treatment which supports the transferability of this population during the first year after curative intent PC treatment. One limitation of the study may be the variation in the civil status of the study population, as few patients were single and men without partners experience higher symptom burden and require more support [59].

The Covid-19 pandemic impacted one patient’s attendance at the initial physical meeting due to fear of exposure. On the other hand, the pandemic led to an increased use of telemedicine and remote monitoring technologies, which probably influenced the retention rate positively in the study.

Moreover, conducting these types of studies in a primary care setting can be considered a strength, given the escalating demands on healthcare providers and the highlighted role of primary care in addressing the needs of the growing cancer population. However, it could be a limitation that the nurse was well-informed and knowledgeable, as it is not common for district nurses to provide such support, and therefore, it may not be representative of the knowledge and experiences of nurses in primary care generally. An inexperienced nurse may have provided a different perspective and yielded information about potential knowledge gaps among nurses in primary care. This was, however, not the main aim of the study. Furthermore, the nurse was also a member of the research team which could have influenced the results of the intervention. However, the study nurse was not involved in the interviews or analysis of the field notes and a strength is that it offers an insider’s perspective that could enrich the intervention’s development. The health dialogues addressed different issues (high alcohol consumption, mental illness) not related to prostate cancer. However, the information was documented in the patient’s health record and not in the field notes which is a limitation as this information could not be analysed. Additionally, we didn’t have ethical approval to analyse health records. It is important in future study design to include information on all communications in health dialogues.

## Conclusion

The patients presented a high level of engagement with the intervention, displaying good adherence and acceptability by utilising the app and its features, and participating in health dialogues with a nurse who had knowledge in oncology and primary care. The combination of the app and health dialogues delivered flexible and tailored support and information, which seemed to increase the patients’ knowledge and awareness of their symptoms. However, some areas for improvement were identified, such as simplifying the log-in procedure. This study demonstrated that the intervention was feasible and acceptable for patients with prostate cancer in primary care settings, and that it had the potential to improve their symptom management. This study supports the testing of the intervention in a larger study in the primary care setting.

## Data Availability

The datasets used and/or analyzed during the current study are available from the corresponding author upon reasonable request.

## Abbreviations

PC: Prostate cancer
PSA: Prostate specific antigen
ePRO: Electronic patient reported outcomes
PCC: Primary care centres
